# Histopathological Features and Protein Markers of Arrhythmogenic Cardiomyopathy

**DOI:** 10.3389/fcvm.2021.746321

**Published:** 2021-12-07

**Authors:** Carlos Bueno-Beti, Angeliki Asimaki

**Affiliations:** Molecular and Clinical Sciences Research Institute, St. George's University of London, London, United Kingdom

**Keywords:** arrhythmogenic cardiomyopathy, sudden cardiac death, desmosomes, intercalated disk, histopathology, protein markers, buccal cells, plasma auto-antibodies

## Abstract

Arrhythmogenic cardiomyopathy (ACM) is a heritable heart muscle disease characterized by syncope, palpitations, ventricular arrhythmias and sudden cardiac death (SCD) especially in young individuals. It is estimated to affect 1:5,000 individuals in the general population, with >60% of patients bearing one or more mutations in genes coding for desmosomal proteins. Desmosomes are intercellular adhesion junctions, which in cardiac myocytes reside within the intercalated disks (IDs), the areas of mechanical and electrical cell-cell coupling. Histologically, ACM is characterized by fibrofatty replacement of cardiac myocytes predominantly in the right ventricular free wall though left ventricular and biventricular forms have also been described. The disease is characterized by age-related progression, vast phenotypic manifestation and incomplete penetrance, making proband diagnosis and risk stratification of family members particularly challenging. Key protein redistribution at the IDs may represent a specific diagnostic marker but its applicability is still limited by the need for a myocardial sample. Specific markers of ACM in surrogate tissues, such as the blood and the buccal epithelium, may represent a non-invasive, safe and inexpensive alternative for diagnosis and cascade screening. In this review, we shall cover the most relevant biomarkers so far reported and discuss their potential impact on the diagnosis, prognosis and management of ACM.

## Introduction

### Demographics

Current experts in the field estimate that ACM affects 1:5,000 individuals in the general population although regional differences exist (1). SCD is often the first manifestation of ACM, and the diagnosis is missed at autopsy, particularly if this is not performed by an expert cardiac pathologist. The disease is also frequently missed clinically, owing to its vast phenotypic manifestation, age-related progression, incomplete penetrance and overlap with other disease entities (1). In a large cohort of ACM patients, the mean age at first evaluation was 36 ± 14 years with a median age of cardiac arrest of 25 years (1). Accounting for up to 20% of the cases, ACM is one of the leading causes of SCD in the young (2) and it is responsible between 4.7 and 27% of SCD in athletes (3–5). The incidence and severity of ACM is higher in men than in women (male/female ratio 2.7:1) (6–8). The pathophysiology behind this difference could be attributed to a direct effect of sex hormones on the disease phenotype or to differences in the amount and intensity of exercise between genders (9, 10).

### Clinical Presentation, Progression, and Diagnosis

The most common clinical manifestations of ACM are syncope, palpitations and SCD caused by ventricular arrhythmias. In the natural history of the classical right ventricular (RV) disease, four different stages have been documented (11). In the first phase (so-called pre-clinical or concealed), patients are at risk of SCD, especially during strenuous exertion, even if structural abnormalities are very subtle or totally absent. The second phase is characterized by an overt electrical disorder with ECG abnormalities such as inverted T-waves and arrhythmias with left bundle branch block morphology. Structural abnormalities are discernible by conventional imaging but restricted to the RV. In the third phase, the extension of the disease through the RV results in isolated right heart failure. Localized involvement of the left ventricle (LV) may occur at this stage but the function of the left heart is still preserved. In the final phase, LV involvement leads to end-stage heart failure with biventricular involvement (11). It seems plausible that during the early stages of the disease, arrhythmias may arise entirely in the context of molecular and subcellular abnormalities. Conversely, at later stages, tissue scarring and fibrofatty replacement of healthy myocardium appears to be responsible for the generation of the arrhythmogenic substrate.

New cardiac imaging techniques and genotype-phenotype correlations identified patients with biventricular and left-dominant form of the disease (11, 12). The most common presentation of these forms are ventricular arrhythmias with right-bundle-branch-block morphology indicating a LV origin and ECG abnormalities such as low QSR voltages in the limb leads and negative T-waves in the lateral or inferolateral leads. LV systolic function is normal or mildly reduced with mild or no dilatation (13). In the left-dominant ACM structural remodeling is found earlier and predominantly in the LV affecting the posterolateral region of LV free wall and, less commonly, the septum (14). Structural remodeling of the LV is most likely responsible for the generation of the arrhythmogenic substrate. In fact, in a recent study, the presence of fat infiltration in the subepicardial posterolateral region of the LV determined by cardiac magnetic resonance supports the diagnosis of left-dominant form of the disease and rules out myocarditis, a known phenocopy of ACM (15).

ACM is a complex disease, and its phenotype is determined by the presence of abnormal electrical and structural substrates. Its diagnosis can be challenging and requires evidence of all structural, functional, and electrophysiological abnormalities. Accordingly, the International Task Force (ITF) criteria for the clinical diagnosis of ACM were proposed for the first time in 1994 (16) and updated in 2010 (17). These include major and minor criteria from six different categories including: repolarization/depolarization abnormalities, arrhythmias, morphological alterations, functional changes, histopathological changes, and family history/genetic findings. Definite ACM diagnosis requires fulfillment of two major criteria, one major and two minor criteria or four minor criteria from different categories. These criteria, however, were more tailored to recognizing the “classical,” RV form of the disease. To improve the diagnosis of left-sided phenotypes, a revision of the 2010 ITF criteria (18) and the introduction of new diagnostic criteria regarding tissue characterization and ventricular arrhythmia features have recently been suggested, resulting in a new set of criteria, The Padua Criteria (13).

Several diseases may mimic ACM making its diagnosis even more challenging. Early phases of ACM are often misdiagnosed as idiopathic right ventricular outflow tract (RVOT) tachycardia (19) or Brugada syndrome (20). In the more advanced biventricular form of the disease, ACM is indistinguishable from dilated cardiomyopathy (DCM) (21). In some instances, patients with cardiac sarcoidosis present with clinical manifestations highly reminiscent of ACM and differential diagnosis is achieved only by the presence of non-caseating granulomas or other sarcoid features not seen in ACM. Other diseases that mimic ACM are myocarditis and pulmonary hypertension (21).

### Genetics

On the Greek island of Naxos, *Protonotarios* and colleagues first described a syndromic form of ACM characterized by cardiomyopathy, wooly hair and palmoplantar keratoderma (PPK). The syndrome, named Naxos disease, was inherited in an autosomal recessive manner and was fully penetrant by adolescence (22). In the year 2000, genetic linkage analysis identified a homozygous deletion in the *JUP* gene (*PG*; encoding for plakoglobin) as the cause of Naxos disease (23). Almost at the same time, *Carvajal-Huerta* and colleagues reported a homozygous truncating mutation in the *DSP* gene (encoding for desmoplakin) as causative of Carvajal Syndrome; a similar disease, characterized by biventricular cardiomyopathy, wooly hair, and PPK (24). Mutations following an autosomal dominant inheritance pattern have also been described for JUP and DSP genes (25, 26). Both plakoglobin and desmoplakin are integral proteins of the desmosome, a specialized adhesion protein complex located at intercellular junctions. In tissues subjected to increased mechanical stress, such as the heart and the epidermis, desmosomes are responsible for maintaining tissue integrity by serving as a mechanical link between the intermediate filaments of two adjacent cells (23, 24).

Genetic studies of other components of the cardiac desmosome in ACM patients led to the discovery of missense and truncating mutations in further desmosomal genes, specifically those coding for: plakophilin 2 (*PKP2*) (27), desmoglein-2 (*DSG2*) (28–30) and desmocollin-2 (*DSC2*) (31–33). Today it is estimated that >60% of ACM patients are bearing one or more mutations in these 5 genes; *PKP2* being the most highly mutated (1, 34). Most commonly, desmosomal mutations follow an autosomal dominant pattern of inheritance with age-related, incomplete penetrance and variable expressivity, although autosomal recessive patterns of inheritance have also been observed such as in Naxos disease and Carvajal Syndrome. The occurrence of ACM patients harboring multiple mutations (compound or digenic heterozygosity) is common and increases the risk of arrhythmias and SCD (1, 10, 35). Importantly, genetic studies to identify an underlying mutation in a proband diagnosed with ACM can aid cascade screening. Mutation-carrying relatives have an earlier onset of symptoms, increased risk of arrhythmias and a 6-fold increased risk of ACM diagnosis compared to relatives without a mutation (36).

Historically, ACM caused by desmosomal mutations has largely been associated to the classical RV variant of the disease. However, exceptions to this general trend have been observed. Patients carrying mutations in PKP2 gene often present LV involvement at advanced stages of the disease while mutations in DSG2 and DSC are often associated to biventricular forms of ACM. Mutations in DSP (37, 38) and more recently in DSG2 (39) genes have been associated to left-dominant forms of the disease.

At the IDs, the desmosomes, together with the adherens and gap junctions, control the electrical and mechanical coupling of cardiomyocytes (40). The tight structural and functional interaction between these macromolecular structures stimulated the search for gene mutations in other constituents of the IDs leading to the discovery of mutations in the adherens junction genes coding for Cadherin 2 (*CDH2*) (41–43) and catenin-α3 (*CTNNA3*) (44).

Mutations in non-desmosomal genes are increasingly recognized and are usually associated with more severe presentation and left-dominant or biventricular forms of ACM. Some of these mutations are in genes encoding for proteins of the cytoskeleton. Patients with mutations in desmin (DES) (45) and filamin C (FLNC) (46) genes have been found to present circumferential pattern of subepicardial late gadolinium enhancement and fibrosis in the LV that was associated with higher risk of SCD (47). Another gene coding for cytoskeleton proteins commonly mutated is titin (TTN) (48). Of note is the fully penetrant mutation (p.S358L) in transmembrane protein 43 (TMEM43) gene responsible for the most aggressive heterozygous form of ACM (type V). Mutation carriers show increased incidence of SCD that is higher in males and common LV involvement (49). Other implicated genes in ACM code for calcium handling proteins such as phospholamban (PLN) (50). Patients carrying the mutation PLN-p.Arg14del present late gadolinium enhancement in the LV posterolateral wall and LV dysfunction. Finally, rare ACM-causing mutations have been identified in the genes coding for the major subunit of the cardiac sodium channel nav1.5 (*SCN5A*) (51) and the profibrotic cytokine transforming growth factor-β3 (*TGFB3*) (52). The frequency of non-desmosomal mutations tends to be very low in large cohorts of ACM patients. However, some of these are found in higher frequencies in specific regions. For instance, the R14del mutation in the *PLN* gene has a very high frequency in the Netherlands due to a founder effect (50, 53). A summary of all genes implicated in ACM pathogenesis can be found in Table 1. Although ACM is a genetically determined cardiomyopathy its relatively late onset, around the second and the fourth decades of life, is still poorly understood and it is believed to be associated with chronic, accumulated stress to the heart. Pathogenic mutations in the genes presented in Table 1 predispose to ACM and environmental factors such as exercise (9, 55, 56) and male gender (57, 58) modulate disease onset and progression.

**Table 1:** Genes associated with ACM.

**Localization/** **function**	**Gene symbol**	**Protein**	**Estimated frequency (%)**	**Mode of inheritance**	**Overlapping diseases**	**Affected ventricle**	**References**
Desmosome	*PKP2*	Plakophilin 2	46	AD	BrS	RV, biventricular	(1, 30)
	*DSP*	Desmoplakin	14	AD, AR (Carvajal Syndrome)	DCM	LV, biventricular	(26, 30)
	*DSG2*	Desmoglein 2	10	AD	DCM	RV, biventricular	(28, 30)
	*DSC2*	Desmocollin 2	9	AD, AR (no skin manifestation)	–	RV, biventricular	(30)
	*JUP*	Plakoglobin	0.4	AD, AR (Naxos disease)	–	RV, biventricular	(1, 30)
Area Composita	*CTNNA3*	Catenin-α3	2.6	AD	–	RV, biventricular	(44)
	*CDH2*	Cadherin 2	1.2	AD	–	RV, biventricular	(41)
Cytoskeleton	*DES*	Desmin	Rare	AD	DCM, HCM	LV, biventricular	(45)
	*FLNC*	Filamin C	Rare	AD	DCM	LV	(46)
	*TMEM43*	transmembrane protein 43	Rare	AD	–	RV, biventricular	(54)
	*TTN*	Titin	Rare	AD	DCM, HCM	RV, LV, biventricular	(48)
Ion transport	*SCN5A*	Nav1.5	Rare	AD	BrS, LQTS	LV, biventricular	(51)
	*PLN*	Phospholamban	Rare	AD	DCM	LV, biventricular	(50)
Cytokine	*TGFB3*	Transforming growth factor-ß3	Rare	AD	–	RV	(52)

*AD, autosomal dominant; AR, autosomal recessive; BrS, Brugada Syndrome; DCM, dilated cardiomyopathy; HCM, hypertrophic cardiomyopathy; LQTS, Long QT syndrome; LV, left ventricle; Nav1.5, α-subunit of the cardiac sodium channel complex; RV, right ventricle*.

Very recently an evidence-based re-evaluation of all reported ACM genes by using the semiquantitative Clinical Genome Resource framework revealed that only 8 of the aforementioned genes (*PKP2, DSP, DSG2, DSC2, JUP/PG, TMEM43, PLN*, and *DES*) had definitive or moderate evidence for ACM (59). Moreover, these genes account for virtually all (97.4%) pathogenic/likely pathogenic ACM variants in Clinvar. Accordingly, the authors recommend that only pathogenic/likely pathogenic alterations in these 8 genes should yield a major criterion for ACM diagnosis (59).

This is crucial, as incomplete/misleading information of the genetics underlying the pathogenesis of ACM can increase the risk of misdiagnosis. Currently, molecular genetic testing is indicated to identify a pathogenic or likely pathogenic mutation in a proband fulfilling the diagnostic criteria for ACM (60). If a mutation is found in the proband, mutation-specific genetic testing is then applied to family members to identify individuals carrying the mutation(s) and thus guide their management accordingly (18).

## Histopathological Features and Protein Markers

### Gross Histological Features

**Figure 1:**
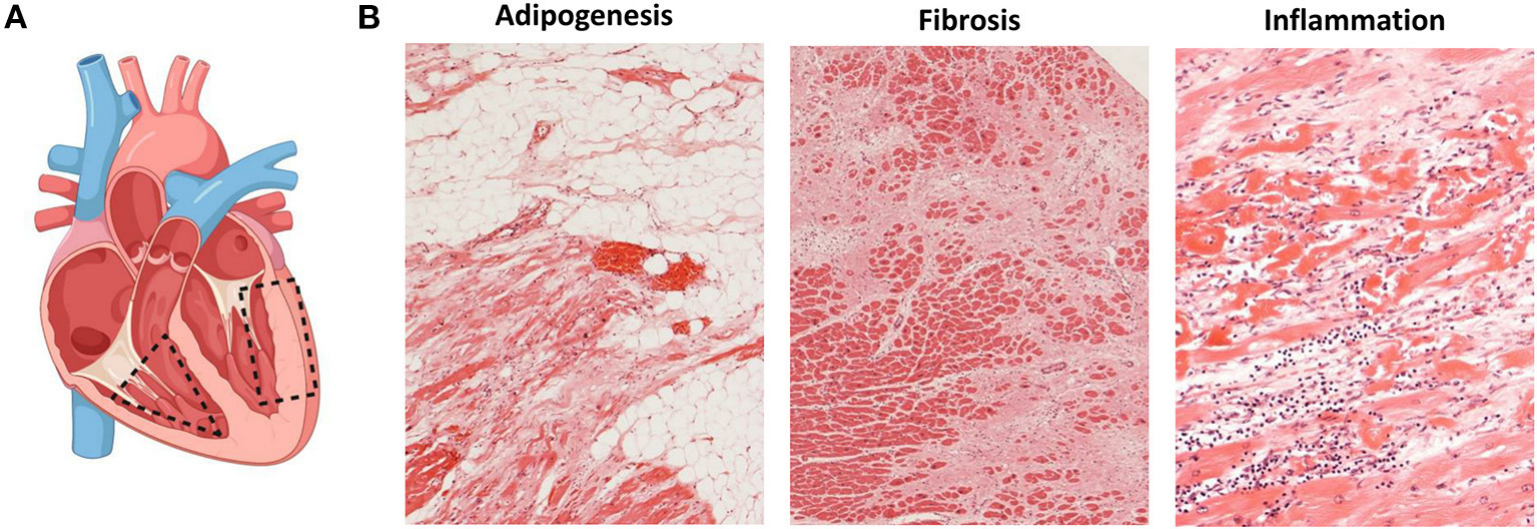
Histopathological changes in ACM. **(A)** Most affected heart regions in ACM. In the right ventricle, structural changes mostly affect areas of the inflow tract, the outflow tract and the apex known as the triangle of dysplasia (dashed triangle). When the left ventricle is affected, structural changes are observed in the inferior and inferolateral walls (dashed rectangle); created with BioRender.com. **(B)** Histopathological changes observed in ACM namely adipogenesis (left), fibrosis (middle) and presence of inflammatory infiltrates (right).

The histological hallmark of ACM is regarded to be the fibrofatty replacement of myocardial tissue associated with ventricular atrophy (2, 6). The pathological changes in the heart start in the epicardium spreading to the endocardium to eventually become transmural in more advanced stages of the disease, mainly affecting the RV free wall. Within the RV, the most common areas affected are the inflow tract, the outflow tract and the apex, collectively known as the triangle of dysplasia (Figure 1A) (6). The endocardial trabeculated muscles of the RV and the septum are generally spared of histopathological changes. Aneurysms, if present, are commonly located in the inflow and outflow tract of the RV. In an autopsy series of classical right-sided cases of ACM, alterations in the LV were found in 76% of the hearts.

Genotype-phenotype correlations and the use of contrast-enhancement cardiac magnetic resonance to evidence fibrofatty replacement in the LV have uncovered biventricular and left-dominant forms of the disease (11, 12). In the left dominant form of ACM, pathohistological structural remodeling can be observed originating in the epicardium and progressing toward the endocardium, to finally become transmural with localized or extensive wall thinning in the most severe presentation of the disease. The fibrofatty replacement is mostly located in the subepicardial inferolateral LV free wall (11, 12, 14). In a large cohort of SCD victims due to ACM, isolated LV disease was observed in 17% whereas biventricular involvement was observed in 70% of the cases. In this cohort, the most common areas of the LV presenting fibrofatty replacement were the posterobasal (68%) and anterolateral walls (58%) (61). Similarly to the classical ACM, structural remodeling of the LV is most likely responsible for the generation of the arrhythmogenic substrate.

The isolated infiltration of epicardial adipocytes is not enough to diagnose ACM as it can be a normal finding in elderly and obese individuals. It must be accompanied by myocyte degeneration and fibrotic replacement (Figure 1B) (62). The current ITF criteria now establish a residual number of cardiomyocytes at <60% by morphometric analysis coupled with fibrous replacement as a major histological criterion (17). The histopathological characterization of fibrofatty replacement of the healthy myocardium can be made either in full hearts (post-mortem or following cardiac transplantation) or in cardiac biopsy samples (17).

The presence of inflammatory infiltrates is another common feature of ACM and it has been reported in up to 75% of hearts at autopsy (Figure 1B) (63). Such infiltrates consist of concentrations of mononuclear cells (lymphocytes and macrophages) around necrotic or injured cardiomyocytes and can be found both in the ventricular walls and the septum (64). The presence of inflammatory infiltrates is indicative of ongoing myocardial damage suggesting a pathological role for inflammation and/or myocarditis in ACM (64). It is not clear yet, however, if the infiltrates accumulate in the heart as a response to myocyte damage of if those cells themselves promote myocyte injury, fibrofatty replacement and arrhythmias through immune mechanisms. Noteworthily, the activation of the major inflammatory pathway NFκB was recently reported as a driver of key features of ACM in several experimental models (65).

Similar to the clinical, natural history of the disease, there are also four histological stages characterizing ACM progression (66). The concealed phase is characterized by minimal or no histological changes. The second phase is characterized by minor histological alterations confined to the RV. The third phase is characterized by extensive RV remodeling with severe dilatation but preserved LV structure/function. Finally, extensive remodeling of both ventricles is evident in advanced disease (67).

### Endomyocardial Biopsies

Given the severe, extended histological changes observed in explanted hearts of ACM patients (Figure 1B) the frequency of false negative results obtained on endomyocardial biopsies (EMBs) is surprising. This can be explained by the natural course of the disease, its patchy nature as well as technical limitations of the sampling process. Since the disease progresses from the epicardium to the endocardium, samples obtained from the endocardium of patients with early/mild forms of the disease may be totally devoid of the aforementioned pathological features (62). Moreover, EMBs tend to be taken from the interventricular septum, generally spared of the histopathological changes of ACM. To determine what area of the heart is more informative for the diagnosis of ACM, Basso et al. analyzed simulated biopsies obtained from various locations of explanted hearts (62). With a specificity of 95% and sensitivity of 80%, the main diagnostic parameter was the amount of residual myocardium (<59%) with the presence of fat (>22%) and fibrous tissue (>31%). The diagnostic yield varied across the different regions of the heart with the “triangle of dysplasia” being the most informative region and the septum and the LV the least (62). Noteworthily, however, sampling the RV free wall comes with an increased risk of ventricular perforation and tamponade (68).

The diagnostic yield of EMBs can be improved by the use of electroanatomic voltage mapping as areas showing low voltage have been associated with myocyte degeneration and fibrofatty replacement. In one study, electroanatomic voltage mapping-guided EBM was diagnostic of ACM in 81% of the cases rendering a high diagnostic sensitivity (69). In another case series, the approach allowed for the accurate differential diagnosis of myocarditis, a known phenocopy of ACM (70). In a very recent study, electroanatomic voltage mapping-guided EMB improved the 2010 ITF criteria diagnostic yield by upgrading one-third of the patients at risk to definite ACM with negligible complications. This study supports the notion that electroanatomic voltage mapping-guided EMB may still be a safe and useful tool for the diagnosis of ACM (71).

Nevertheless, EMB is still considered an invasive procedure with associated risks that has a low diagnostic yield. Nowadays, it is rarely performed in the initial diagnosis of the disease. It is indicated in cases of non-familial ACM for differential diagnosis of phenocopies (72), specifically in probands with sporadic ACM and negative gene testing to exclude sarcoidosis, myocarditis, or other heart muscle disorders (13). Post-mortem and explanted hearts do not have any of these limitations and should be meticulously examined by expert cardiac pathologists whenever possible (73). Immunohistochemical examination of protein markers of ACM in heart samples or surrogate tissues may provide added diagnostic value and are reviewed in the next section.

### Protein Markers in Heart Tissue

The discovery of the first ACM-causative genes, has helped the scientific community uncover some of the mechanisms driving the pathophysiology of the disease. As a result, key molecular players were identified, which can be used in the future as therapeutic targets and/or specific molecular markers of the disease. In this section we will cover the protein markers in heart tissue samples reported so far that can potentially help the diagnosis and risk stratification of ACM.

Human myocardial samples obtained at autopsy or following transplantation are most commonly used in the search of specific protein markers. Due to the associated risk of RV perforation and tamponade, the studies on EMBs have been more limited. Following the discovery of the causative gene for Naxos disease (23), the most logical candidate to investigate first was plakoglobin. In 2004 Kaplan et al. showed for the first time that even if the mutated plakoglobin was expressed in the hearts of Naxos disease patients it failed to reach the IDs where the protein executes its structural roles (74). The re-distribution of plakoglobin was accompanied by gap junction remodeling as evidenced by decreased immunoreactive signal for Connexin 43 (*Cx43*; the major ventricular gap junction protein) at the IDs and smaller/fewer gap junctions connecting ventricular myocytes in both RV and LV samples. The expression of other desmosomal proteins at the IDs of patients with Naxos disease was normal (74). In a patient with Carvajal Syndrome, similar findings were reported. In this case, it was desmoplakin, being the mutated protein, that failed to localize at the IDs (75). Gap junction remodeling was also observed in this syndrome. Interestingly, the localization of plakoglobin at the ID was also prevented, even if it was not itself mutated (75). This observation revealed for the first time, that a mutation in a gene coding for a given protein can affect the localization of further, interacting proteins, even if they are not themselves affected by genetic alterations.

**Figure 2:**
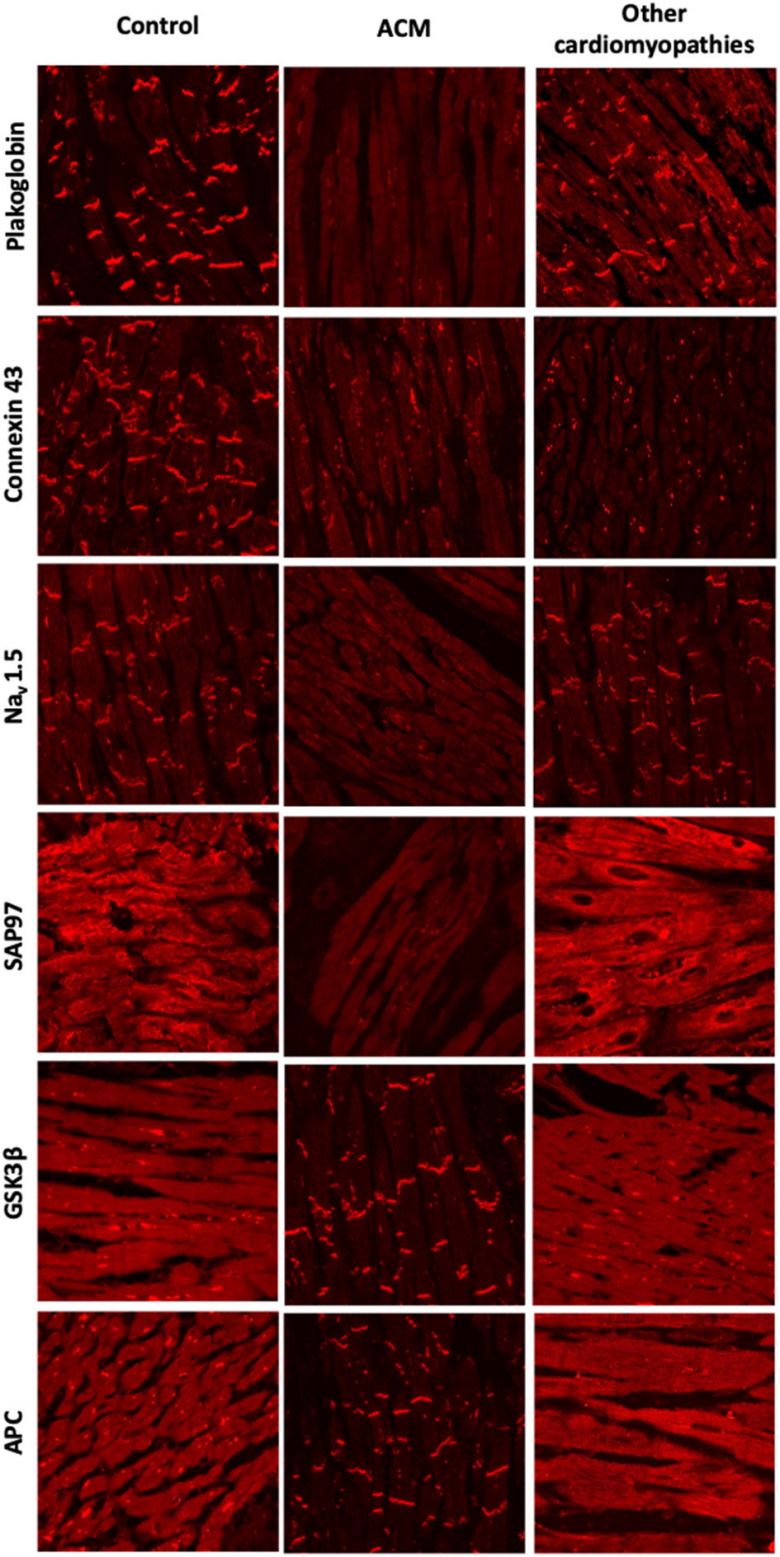
Protein re-distribution in the hearts of ACM patients. Representative confocal immunofluorescence images showing loss of junctional signal for PG, Cx43, and Nav1.5 in ACM myocardium compared to controls. Additionally, both sarcomeric and junctional signal for SAP97 is lost while GSK3β and APC translocate to the junctions. Myocardium from patients with other forms of cardiomyopathy show normal distribution of PG, Nav1.5, GSK3β and APC. Gap junction remodeling is, however, evident and ID signal for SAP97 is also lost, though its sarcomeric distribution is preserved.

These findings were validated in larger cohorts of patients with a definite diagnosis of ACM and dominantly inherited mutations in the *DSP, DSC2, PKP2*, or *DSG2* genes. Most patients showed a marked reduction in immunoreactive signal for plakoglobin at the ID, which was not confined to areas of the RV showing pathological changes but was also present in areas of the LV and septum that appeared structurally normal (Figure 2). This observation suggested that even an EMB sample obtained from the right side of the septum would show this diagnostic change hence reducing the number of false negatives and risk associated with this technique. Other desmosomal proteins showed variable patterns of distribution confirming that mutations in a single protein can affect the localization of other non-mutated partners. Plakoglobin redistribution from the ID to the cytosolic pool was shown to be specific for ACM as this change was not observed in heart samples of patients with documented hypertrophic cardiomyopathy (HCM), DCM or ischemic cardiomyopathy (ICM) (76). It is important to highlight that in order to bring up differences in PG junctional distribution between control and ACM myocardial samples a broad range of antibody dilutions need to be tested. The endpoint is to achieve a binary result up to the point where you detect signal or no signal at the intercalated disks of the cardiomyocytes. By removing the necessity of image quantification, we reduce the subjectivity in the interpretation of the results while increasing the reproducibility of the technique across laboratories (77).

Cx43 expression was diffusely reduced throughout the heart in all ACM samples analyzed in this cohort but was not specific of ACM. Reduced Cx43 expression at the IDs has been observed in end-stage HCM, DCM, and ICM, being more apparent in areas with substantial structural remodeling (78, 79). However, in ACM, gap junction remodeling seems to occur diffusely in early stages of the disease in areas of the heart with minimal or no structural remodeling, potentially playing a primary role in the highly arrhythmogenic nature of this disease (76). How mutations in desmosomal proteins can impact on the stability and function of the gap junctions it is poorly understood. More recently, the molecular mechanism behind gap junction remodeling in ACM has been uncovered by using a murine model of desmosomal ACM. Specifically, it has been shown that DSP maintains the integrity of the gap junctions by inhibiting Connexin 43 lysosomal degradation (80). Despite its specificity among the cardiomyopathies, however, plakoglobin redistribution could not discriminate ACM from sarcoidosis and giant cell myocarditis (81). Plakoglobin labeling is an additional test but on its own it is not conclusive for the diagnosis of ACM. For this reason, is has not been added to the ITF criteria as of now.

Furthermore, EMB samples obtained from two patients with subclinical ACM bearing *DSP* variants showed loss of ID immunoreactive signal for desmoplakin but not for plakoglobin (82). Notably, *DSP* variants have been increasingly linked to biventricular or left-dominant ACM. In fact, a recent report, suggested that patients bearing pathogenic *DSP* variants manifest with a disease distinct from ACM or DCM, termed desmoplakin cardiomyopathy (37, 38).

Reduced densities of the cardiac I_Na_ current and the inward rectifying K^+^ current I_K1_ have been reported in different experimental models of ACM (83–85). Moreover, induced pluripotent stem cell-derived cardiomyocytes (iPSC-CMs) from a patient bearing a mutation in *PKP2* showed reduced I_Na_ (86) and abnormal Ca^+2^ dynamics (87). There is also direct evidence of reduced ID immunoreactive signal for Nav1.5 in patients with ACM (77). These electrophysiological alterations together with gap junctional remodeling could be responsible for the highly arrhythmogenic nature of ACM at its early stages. One possible explanation for these observations is that mutations in desmosomal proteins could alter the complex interactions that take place within the ID between the mechanical, electrical and gap junction components. However, both Nav1.5 and Kir2.1 (the major protein responsible for the I_K1_ current) have SIV motifs binding to the PDZ domains of the synapse-associated protein 97 (SAP97). SAP97 silencing seems to regulate Nav1.5 and the stoichiometry of Nav1.5 and Kir2.1 at the ID (88, 89). Also, SAP97 together with the ion channel proteins traffic as a multiprotein complex, suggesting a role for abnormal protein trafficking in ACM. Decreased immunoreactive signal for SAP97 was observed at the IDs in the myocardium of patients with ACM as well as two *in vitro* models of the disease (85). Sarcomeric signal for SAP97 was retained in the myocardium of patients with HCM, DCM or ICM although concentrated ID signal was also lost. Accordingly, loss of immunoreactive signal from both sarcomeric and junctional pools appears to be a specific feature of ACM (Figure 2) (85).

A drug screening study in a transgenic zebrafish model of ACM revealed that one compound, SB216763, a specific inhibitor of glycogen synthase kinase 3β (GSK3β), prevented all disease endpoints in this experimental model (85). Moreover, in two transgenic mouse models of ACM, SB216763 was able to prevent all clinical and subcellular disease features (90). In agreement with the work led by professor Saffitz, more recently Padrón-Barthe et al. have recapitulated these results in another *in vitro* (iPS) and *in vivo* (transgenic mouse) experimental models of non-desmosomal type 5 ACM caused by the expression of TMEM43 p.S358L mutation (91). In the healthy myocardium, GSK3β and its binding partner adenomatous polyposis coli (APC) show a diffuse cytoplasmic localization. In sharp contrast, in ACM myocardium, the proteins strongly localize at the IDs. GSK3β and APC redistribution from the cytosol to the ID was specific for ACM as it was not observed in other forms of heart disease including HCM, DCM, ICM, or cardiac sarcoidosis (Figure 2) (85).

Protein redistribution in the hearts of ACM patients occurs diffusely throughout the myocardium, preceding gross histopathologic changes. EMBs from the right side of the septum would thus show this diagnostic change, increasing the diagnostic yield of this technique. Not all the protein changes presented in this section are specific to ACM. Their combination, however, may represent an unequivocal molecular signature for the disease. Redistribution of plakoglobin, Cx43, Nav1.5, and SAP97 from the ID to the cytoplasm in conjunction with a shift of GSK3β and APC from the cytosol to the ID appears to be the most consistent finding in the myocardium of most ACM patients analyzed to date (Figure 2). More recently, it has been found that in non-desmosomal ACM caused by the PLN p.Arg14del mutation, key protein distribution in the heart differs to that exhibited by classical, desmosomal ACM (92). This indicates that additional genetic variants may contribute to the phenotypical heterogeneity of ACM.

Highly informative as this “molecular signature” may be, its use is still limited by the implicit need for a heart sample. As mentioned above, EMBs are invasive and risky, tend to be used as a “last resort” and could not be used to screen potentially healthy family members of ACM patients. This limitation would be bypassed if similar diagnostic information could be obtained from surrogate tissues expressing desmosomal proteins such as the hair follicles or the skin, particularly the buccal epithelium; a specialized form of skin that does not require a full thickness biopsy procedure to be sampled. Some groups have explored this idea and their findings are covered in the next section.

### Protein Markers in Buccal Mucosa Cells

The buccal mucosa consists of non-keratinized stratified squamous epithelium where cells adhere to one another through different types of junctional structures. Of these structures, desmosomes are the ones connecting the keratin intermediate filaments of adjacent cells creating a 3D array within the entire epithelium (93). The protein makeup of a desmosome is very similar between the buccal mucosa and the heart. Buccal cells can be obtained easily and safely through a non-invasive procedure and therefore they would represent the ideal surrogate tissue to study protein redistribution in ACM patients and unaffected family members at a relatively low cost.

Accordingly, the localization of plakoglobin, desmoplakin, plakophilin-1 (*PKP1*; an isoform of PKP2 expressed in the upper epithelia) and Cx43 was investigated in the buccal mucosa of 39 patients with a definite clinical diagnosis of ACM bearing desmosomal gene mutations. Protein localization was also investigated in 15 family members of the aforementioned probands, who were bearing ACM-causing variants without showing clinical evidence of disease. Buccal smears from 40 individuals with no family history or clinical evidence of heart disease served as negative controls. Cells from 7 individuals with other forms of cardiomyopathy were used to test the specificity of the findings (94). Immunoreactive signal for plakoglobin and Cx43 was reduced in the buccal mucosa of the majority of ACM patients when compared to healthy controls and individuals with other forms of heart disease. Interestingly, junctional signal for these proteins was also decreased in the majority of family members carrying mutated alleles without showing evidence of heart disease, suggesting that redistribution of desmosomal proteins does not necessarily correlate with clinical expression of the disease.

**Figure 3:**
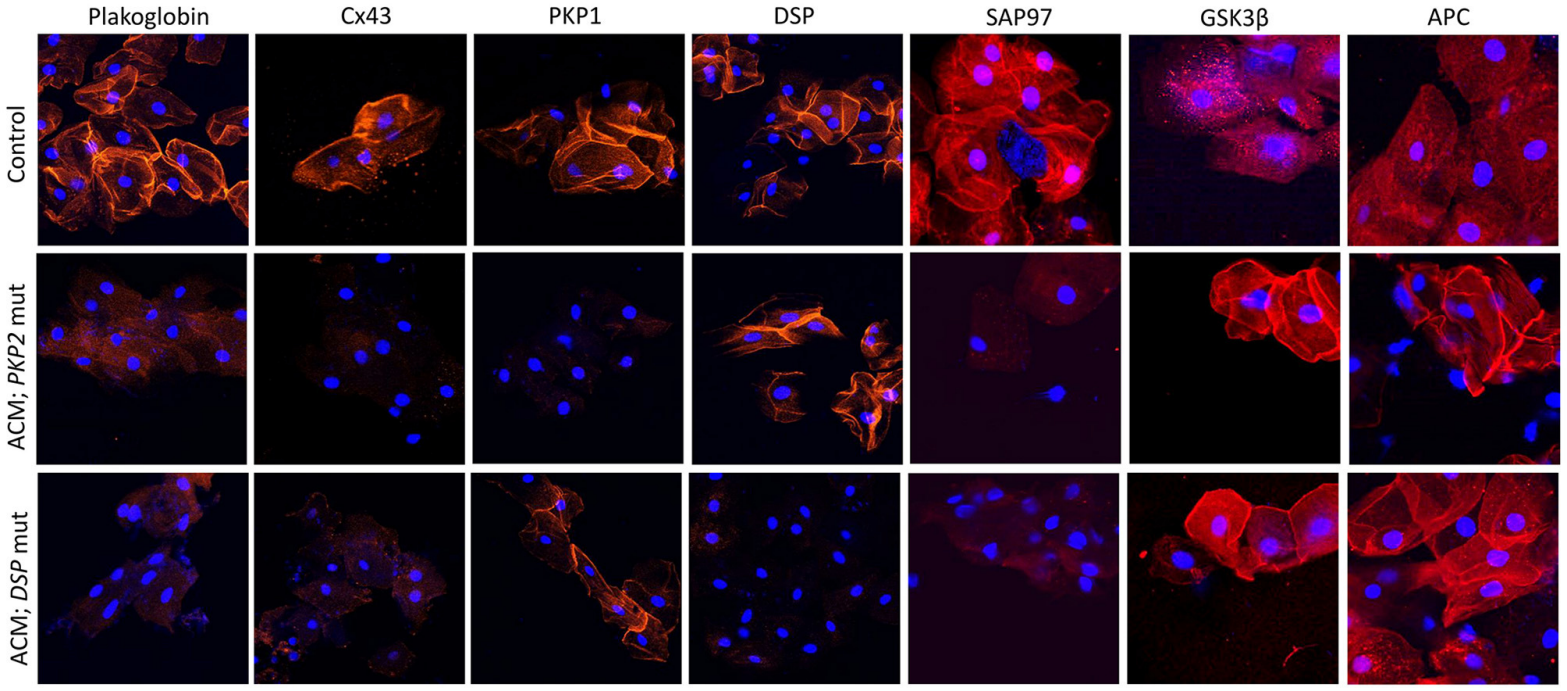
Protein re-distribution in buccal mucosa cells from ACM patients. Representative confocal immunofluorescence images showing loss of PG, Cx43 and SAP97 membrane signal in buccal cells from ACM patients bearing a mutation in *PKP2* or *DSP* compared to control subjects. Conversely, signal for GSK3β and APC shifts from cytosolic to junctional pools. The patient bearing a mutation in *PKP2* shows loss of PKP1 signal but not DSP while the patient bearing a mutation in *DSP* shows loss of DSP signal but not PKP1. Immunoreactive signal of PG, Cx43, PKP1, DSP, SAP97, GSK3β and APC in red; nuclei were counterstained with DAPI in blue.

Another interesting finding in this study was the link between the reduced junctional localization of specific desmosomal proteins in the buccal mucosa cells and the mutant gene of interest. Signal for desmoplakin was reduced in buccal mucosa cells from patients bearing mutations in *DSP, DSC2*, or *DSG2* but not patients bearing mutations in *PKP2*. Similarly, signal for PKP1 was reduced in buccal mucosa cells from patients bearing mutations in *PKP2* but not those with mutations in *DSP, DSC2*, or *DSG2* (Figure 3). This association becomes even more interesting if one considers that PKP1 and PKP2 are expressed by different genes, located on different chromosomes. Yet, they appear to share common regulatory mechanisms where a mutation in one isoform expressed in one tissue, can affect the localization of a different isoform, expressed in a different tissue. Importantly, when buccal mucosa cells of ACM patients were cultured *in vitro* and exposed to SB216763, abnormal protein distribution for plakoglobin and Cx43 was reversed (94). Similarly to what was observed in myocardial tissue samples, in buccal mucosa cells obtained from healthy subjects, there was strong junctional signal for SAP97 and diffuse cytosolic signal for GSK3β and APC. Conversely, buccal cells obtained from ACM subjected showed loss of junctional signal for SAP97 and strong membrane signal for GSK3β and APC. These findings were consistent in all ACM cases regardless of the underlying desmosomal mutation causing the disease (Figure 3).

DCM patients carrying a frameshift mutation in the *FLNC* gene, show normal expression for plakoglobin and reduced expression for DSP, Cx43, and SAP97 in their buccal cells, indicating a partial overlap of the pathological cellular phenotype between desmosomal and non-desmosomal ACM (95). More recently, the initial observation of reduced plakoglobin expression in the buccal mucosa of ACM patients carrying a desmosomal mutation was confirmed in another patient cohort from the Netherlands (96). However, buccal mucosa cells from ACM patients carrying the R14del mutation in *PLN* showed normal plakoglobin expression, pointing at distinct pathological mechanisms (96).

Despite the non-cardiac origin of buccal mucosa cells, they show similar pathologic protein distribution to that exhibited by cardiac myocytes. Buccal mucosa cells could as well represent a new *in vitro* model to study disease mechanisms and aid drug screening for ACM in general and in personalized medicine to monitor the patient's response to a specific treatment. Confirmatory studies in larger cohorts of ACM patients and family members are still needed to validate the buccal mucosa as a source of meaningful information for the diagnosis and risk stratification in ACM.

### Autoantibodies

Antibodies against self-antigens (autoantibodies) are often detected in the plasma of patients with inflammatory diseases such as myocarditis and pemphigus. Due to the complexity of inflammatory diseases, however, the contribution of autoantibodies to disease progression in these conditions is incompletely known. Recently, a few studies showed the potential of autoantibodies in the diagnosis of ACM. Chatterjee et al. evaluated the presence of antibodies against cardiac desmosomal cadherin proteins in the sera of a small cohort of patients with ACM. They identified autoantibodies against DSG2 in the sera of all the patients with ACM, regardless of the genetic background, while the autoantibodies were absent in virtually all the control subjects. The level of anti-DSG2 antibodies correlated with disease burden and caused gap junction dysfunction (97). Whether this marker is specific for the diagnosis of ACM is still to be proven in larger cohorts and in known phenocopies of the disease such as sarcoidosis. The potential of the autoantibodies in detecting subclinical disease in mutation carriers should as well be explored. Caforio et al. found anti-heart antibodies and anti-ID antibodies at a higher frequency in ACM probands, affected relatives and healthy relatives when compared to non-inflammatory cardiac disease, ICM or healthy subjects, providing evidence of autoimmunity in the course of ACM (98). More recently, a combination of four autoantibodies against α-cardiac actin, α-skeletal actin, keratin, and Cx43 has been proposed as a highly sensitive and specific biomarker for Brugada syndrome, irrespective of the underlying genetic cause. These autoantibodies were absent in sera samples from patients with ACM, HCM or DCM (99). Although these findings may have diagnostic potential, extensive confirmatory studies are necessary before autoantibodies can be adopted as a novel diagnostic tool for ACM (100).

## Conclusions

ACM is a complex and progressive disease. SCD may occur in the early stages of the disease in the absence of pathologic structural changes making diagnosis and risk stratification quite challenging. Hearts of SCD victims should be examined by expert cardiac pathologists as a definite post-mortem diagnosis of ACM can greatly aid cascade screening and management of surviving family members. Obtaining EMB samples from patients with equivocal diagnoses is an invasive and risky procedure, and the histological analysis of such samples has a low diagnostic yield. Analysis of localization of key protein markers in the heart can greatly improve the diagnostic yield. However, the utility of this approach is greatly limited by the need for a heart sample. The identification of surrogate tissues mimicking the pathological changes of the heart such the buccal mucosa and the presence of specific autoantibodies in the blood samples of patients with ACM may transform the diagnosis, prognosis, and management of the disease as well as the screening of the patient's relatives that may be at risk of fatal arrhythmias or SCD. More research is needed before these protein markers can be adopted as diagnostic tools for ACM. We are confident, however, that one of these approaches, or a combination of them, will improve the detection, risk stratification and management of ACM reducing the burden of SCD.

## Author Contributions

CB-B prepared the manuscript draft. AA reviewed and approved the draft for submission. All authors contributed to the article and approved the submitted version.

## Funding

AA was supported by the Rosetrees Foundation Trust corn seed fund (M689), the British Heart Foundation project grant (PG/18/27/33616) and the Wellcome Trust project grant (208460/Z/17/Z). CB-B was supported by the British Heart Foundation project grant (PG/18/27/33616).

## Conflict of Interest

The authors declare that the research was conducted in the absence of any commercial or financial relationships that could be construed as a potential conflict of interest.

## Publisher's Note

All claims expressed in this article are solely those of the authors and do not necessarily represent those of their affiliated organizations, or those of the publisher, the editors and the reviewers. Any product that may be evaluated in this article, or claim that may be made by its manufacturer, is not guaranteed or endorsed by the publisher.

## Abbreviations

ACM: Arrhythmogenic Cardiomyopathy
APC: Adenomatous Polyposis Coli
CDH2: Cadherin 2
CTNNA3: Catenin-α3
Cx43: Connexin 43
DCM: Dilated Cardiomyopathy
DCS2: Desmocollin-2
DES: Desmin
DSG2: Desmoglein-2
DSP: Desmoplakin
ECG: Electrocardiogram
EMB: Endomyocardial Biopsies
FLNC: Filamin C
GSK3β: Glycogen Synthase Kinase 3β
HCM: Hypertrophic Cardiomyopathy
ICM: Ischaemic Cardiomyopathy
ID: Intercalated Disk
iPSC-CMs: Induced Pluripotent Stem Cell-derived Cardiomyocytes
ITF: International Task Force
LV: Left Ventricle
PG: Plakoglobin
PKP: Palmoplantar Keratoderma
PKP1: Plakophilin-1
PKP2: Plakophilin 2
PLN: Phospholamban
RV: Right Ventricle
RVOT: Right Ventricular Outflow Tract
SAP97: Synapse-Associated Protein 97
SCD: Sudden Cardiac Death
SCN5A: Cardiac Sodium Channel Nav1. 5
TGFB3: Transforming Growth Factor-β3
TMEM43: Transmembrane protein 43
TTN: Titin.

## References

[1] Groeneweg JA Bhonsale A James CA te Riele AS Dooijes D Tichnell C. Clinical presentation, long-term follow-up, and outcomes of 1001 arrhythmogenic right ventricular dysplasia/cardiomyopathy patients and family members. Circ Cardiovasc Genet. (2015) 8:437–46. 10.1161/CIRCGENETICS.114.001003 25820315

[2] Thiene G Nava A Corrado D Rossi L Pennelli N. Right ventricular cardiomyopathy and sudden death in young people. N Engl J Med. (1988) 318:129–33. 10.1056/NEJM198801213180301 3336399

[3] Finocchiaro G Papadakis M Robertus J-L Dhutia H Steriotis AK Tome M. Etiology of sudden death in sports insights from a United Kingdom regional registry. J Am Coll Cardiol. (2016) 67:2108–15. 10.1016/j.jacc.2016.02.062 27151341

[4] Wasfy MM Hutter AM Weiner RB. Sudden cardiac death in athletes. Methodist Debakey Cardiovasc J. (2016) 12:76–80. 10.14797/mdcj-12-2-76 27486488 PMC4969030

[5] D'Ascenzi F Valentini F Pistoresi S Frascaro F Piu P Cavigli L. Causes of sudden cardiac death in young athletes and non-athletes: systematic review and meta-analysis sudden cardiac death in the young. Trends Cardiovas Med. (2021) 10.1016/j.tcm.2021.06.001. [Epub ahead of print]. 34166791

[6] Marcus FI Fontaine GH Guiraudon G Frank R Laurenceau JL Malergue C. Right ventricular dysplasia: a report of 24 adult cases. Circulation. (1982) 65:384–98. 10.1161/01.CIR.65.2.384 7053899

[7] Hulot J-S Jouven X Empana J-P Frank R Fontaine G. Natural history and risk stratification of arrhythmogenic right ventricular dysplasia/cardiomyopathy. Circulation. (2004) 110:1879–84. 10.1161/01.CIR.0000143375.93288.82 15451782

[8] Choudhary N Tompkins C Polonsky B McNitt S Calkins H Mark Estes NA III. Clinical presentation and outcomes by sex in arrhythmogenic right ventricular cardiomyopathy: findings from the North American ARVC registry. J Cardiovasc Electr. (2016) 27:555–62. 10.1111/jce.12947 26840461 PMC4879587

[9] James CA Bhonsale A Tichnell C Murray B Russell SD Tandri H. Exercise increases age-related penetrance and arrhythmic risk in arrhythmogenic right ventricular dysplasia/cardiomyopathy–associated desmosomal mutation carriers. J Am Coll Cardiol. (2013) 62:1290–7. 10.1016/j.jacc.2013.06.033 23871885 PMC3809992

[10] Rigato I Bauce B Rampazzo A Zorzi A Pilichou K Mazzotti E. Compound and digenic heterozygosity predicts lifetime arrhythmic outcome and sudden cardiac death in desmosomal gene–related arrhythmogenic right ventricular cardiomyopathy. Circ Cardiovasc Genet. (2013) 6:533–42. 10.1161/CIRCGENETICS.113.000288 24070718

[11] Sen-Chowdhry S Syrris P Ward D Asimaki A Sevdalis E McKenna WJ. Clinical and genetic characterization of families with arrhythmogenic right ventricular dysplasia/cardiomyopathy provides novel insights into patterns of disease expression. Circulation. (2007) 115:1710–20. 10.1161/CIRCULATIONAHA.106.660241 17372169

[12] Sen-Chowdhry S Syrris P Prasad SK Hughes SE Merrifield R Ward D. Left-dominant arrhythmogenic cardiomyopathy an under-recognized clinical entity. J Am Coll Cardiol. (2008) 52:2175–87. 10.1016/j.jacc.2008.09.019 19095136

[13] Corrado D Marra Perazzolo M Zorzi A Beffagna G Cipriani A Lazzari MD. Diagnosis of arrhythmogenic cardiomyopathy: the Padua criteria. Int J Cardiol. (2020) 319:106–14. 10.1016/j.ijcard.2020.06.005 32561223

[14] Lazzari MD Zorzi A Cipriani A Susana A Mastella G Rizzo A. Relationship between electrocardiographic findings and cardiac magnetic resonance phenotypes in arrhythmogenic cardiomyopathy. J Am Heart Assoc. (2018) 7:e009855. 10.1161/JAHA.118.009855 30571483 PMC6404435

[15] Andreini D Conte E Casella M Mushtaq S Pontone G Russo AD. Cardiac magnetic resonance features of left dominant arrhythmogenic cardiomyopathy: differential diagnosis with myocarditis. Int J Cardiovasc Imaging. (2021) 1–9. 10.1007/s10554-021-02408-8. [Epub ahead of print]. 34546457

[16] McKenna WJ Thiene G Nava A Fontaliran F Blomstrom-Lundqvist C Fontaine G. Diagnosis of arrhythmogenic right ventricular dysplasia/cardiomyopathy. Task Force of the Working Group Myocardial and Pericardial Disease of the European Society of Cardiology and of the Scientific Council on Cardiomyopathies of the International Society and Federation of Cardiology. Br Heart J. (1994) 71:215–8. 10.1136/hrt.71.3.215 8142187 PMC483655

[17] Marcus FI McKenna WJ Sherrill D Basso C Bauce B Bluemke DA. Diagnosis of arrhythmogenic right ventricular cardiomyopathy/dysplasia: proposed modification of the Task Force Criteria. Eur Heart J. (2010) 31:806–14. 10.1093/eurheartj/ehq025 20172912 PMC2848326

[18] Corrado D van Tintelen PJ McKenna WJ Hauer RNW Anastastakis A Asimaki A. Arrhythmogenic right ventricular cardiomyopathy: evaluation of the current diagnostic criteria and differential diagnosis. Eur Heart J. (2019) 41:1414–29. 10.1093/eurheartj/ehz669 31637441 PMC7138528

[19] Ren L Liu Z Jia Y Fang P Pu J Zhang S. Electrocardiographic difference between ventricular arrhythmias from the right ventricular outflow tract and idiopathic right ventricular arrhythmias. Pacing Clin Electrophysiol. (2014) 37:1658–64. 10.1111/pace.12463 25081355

[20] Agullo-Pascual E Cerrone M Delmar M. Arrhythmogenic cardiomyopathy and Brugada syndrome: diseases of the connexome. Febs Lett. (2014) 588:1322–30. 10.1016/j.febslet.2014.02.008 24548564 PMC3989410

[21] Ellinor PT MacRae CA Thierfelder L. Arrhythmogenic right ventricular cardiomyopathy. Heart Fail Clin. (2010) 6:161–77. 10.1016/j.hfc.2009.12.003 20347785

[22] Protonotarios N Tsatsopoulou A Patsourakos P Alexopoulos D Gezerlis P Simitsis S. Cardiac abnormalities in familial palmoplantar keratosis. Brit Heart J. (1986) 56:321. 10.1136/hrt.56.4.321 2945574 PMC1236865

[23] McKoy G Protonotarios N Crosby A Tsatsopoulou A Anastasakis A Coonar A. Identification of a deletion in plakoglobin in arrhythmogenic right ventricular cardiomyopathy with palmoplantar keratoderma and woolly hair (Naxos disease). Lancet. (2000) 355:2119–24. 10.1016/S0140-6736(00)02379-5 10902626

[24] Norgett EE Hatsell SJ Carvajal-Huerta L Cabezas J-CR Common J Purkis PE. Recessive mutation in desmoplakin disrupts desmoplakin–intermediate filament interactions and causes dilated cardiomyopathy, woolly hair and keratoderma. Hum Mol Genet. (2000) 9:2761–6. 10.1093/hmg/9.18.2761 11063735

[25] Asimaki A Syrris P Wichter T Matthias P Saffitz JE McKenna WJ. A novel dominant mutation in plakoglobin causes arrhythmogenic right ventricular cardiomyopathy. Am J Hum Genet. (2007) 81:964–73. 10.1086/521633 17924338 PMC2265660

[26] Rampazzo A Nava A Malacrida S Beffagna G Bauce B Rossi V. Mutation in human desmoplakin domain binding to plakoglobin causes a dominant form of arrhythmogenic right ventricular cardiomyopathy. Am J Hum Genet. (2002) 71:1200–6. 10.1086/344208 12373648 PMC385098

[27] Gerull B Heuser A Wichter T Paul M Basson CT McDermott DA. Mutations in the desmosomal protein plakophilin-2 are common in arrhythmogenic right ventricular cardiomyopathy. Nat Genet. (2004) 36:1162–4. 10.1038/ng1461 15489853

[28] Pilichou K Nava A Basso C Beffagna G Bauce B Lorenzon A. Mutations in desmoglein-2 gene are associated with arrhythmogenic right ventricular cardiomyopathy. Circulation. (2006) 113:1171–9. 10.1161/CIRCULATIONAHA.105.583674 16505173

[29] Gehmlich K Asimaki A Cahill TJ Ehler E Syrris P Zachara E. Novel missense mutations in exon 15 of desmoglein-2: role of the intracellular cadherin segment in arrhythmogenic right ventricular cardiomyopathy? Heart Rhythm. (2010) 7:1446–53. 10.1016/j.hrthm.2010.08.007 20708101 PMC2994644

[30] Xu Z Zhu W Wang C Huang L Zhou Q Hu J. Genotype-phenotype relationship in patients with arrhythmogenic right ventricular cardiomyopathy caused by desmosomal gene mutations: A systematic review and meta-analysis. Sci Rep. (2017) 7:41387. 10.1038/srep41387 28120905 PMC5264593

[31] Syrris P Ward D Evans A Asimaki A Gandjbakhch E Sen-Chowdhry S. Arrhythmogenic right ventricular dysplasia/cardiomyopathy associated with mutations in the desmosomal gene desmocollin-2. Am J Hum Genet. (2006) 79:978–84. 10.1086/509122 17033975 PMC1698574

[32] Heuser A Plovie ER Ellinor PT Grossmann KS Shin JT Wichter T. Mutant desmocollin-2 causes arrhythmogenic right ventricular cardiomyopathy. Am J Hum Genet. (2006) 79:1081–8. 10.1086/509044 17186466 PMC1698714

[33] Beffagna G Bortoli MD Nava A Salamon M Lorenzon A Zaccolo M. Missense mutations in Desmocollin-2 N-terminus, associated with arrhythmogenic right ventricular cardiomyopathy, affect intracellular localization of desmocollin-2 *in vitro*. BMC Med Genet. (2007) 8:65–5. 10.1186/1471-2350-8-65 17963498 PMC2190757

[34] den Haan AD Tan BY Zikusoka MN Lladó LI Jain R Daly A. Comprehensive desmosome mutation analysis in North Americans with arrhythmogenic right ventricular dysplasia/cardiomyopathy. Circ Cardiovasc Genet. (2009) 2:428–35. 10.1161/CIRCGENETICS.109.858217 20031617 PMC2801867

[35] Xu T Yang Z Vatta M Rampazzo A Beffagna G Pilichou K. Compound and digenic heterozygosity contributes to arrhythmogenic right ventricular cardiomyopathy. J Am Coll Cardiol. (2010) 55:587–97. 10.1016/j.jacc.2009.11.020 20152563 PMC2852685

[36] Cox MGPJ van der Zwaag PA van der Werf C van der Smagt JJ Noorman M Bhuiyan ZA. Arrhythmogenic right ventricular dysplasia/cardiomyopathy. Circulation. (2011) 123:2690–700. 10.1161/CIRCULATIONAHA.110.988287 21606396

[37] Smith ED Lakdawala NK Papoutsidakis N Aubert G Mazzanti A McCanta AC. Desmoplakin cardiomyopathy, a fibrotic and inflammatory form of cardiomyopathy distinct from typical dilated or arrhythmogenic right ventricular cardiomyopathy. Circulation. (2020) 141:1872–84. 10.1161/CIRCULATIONAHA.119.044934 32372669 PMC7286080

[38] Wang W Murray B Tichnell C Gilotra NA Zimmerman SL Gasperetti A. Clinical characteristics and risk stratification of desmoplakin cardiomyopathy. Ep Europace. (2021) euab183. 10.1093/europace/euab183. [Epub ahead of print]. 34352074 PMC8824516

[39] Lao N Laiq Z Courson J Al-Quthami A. Left-dominant arrhythmogenic cardiomyopathy: an association with desmoglein-2 gene mutation—a case report. Eur Hear J Case Rep. (2021) 5:ytab213. 10.1093/ehjcr/ytab213 34263121 PMC8274644

[40] Moncayo-Arlandi J Brugada R. Unmasking the molecular link between arrhythmogenic cardiomyopathy and Brugada syndrome. Nat Rev Cardiol. (2017) 14:744–56. 10.1038/nrcardio.2017.103 28703223

[41] Ghidoni A Elliott PM Syrris P Calkins H James CA Judge DP. Cadherin 2-related arrhythmogenic cardiomyopathy: prevalence and clinical features. Circ Genom Precis Med. (2021) 14:e003097 10.1161/CIRCGEN.120.003097 33566628 PMC8284361

[42] Mayosi BM Fish M Shaboodien G Mastantuono E Kraus S Wieland T. Identification of Cadherin 2 (CDH2) mutations in arrhythmogenic right ventricular cardiomyopathy. Circ Cardiovasc Genet. (2017) 10:e001605. 10.1161/CIRCGENETICS.116.001605 28280076

[43] Turkowski KL Tester DJ Bos JM Haugaa KH Ackerman MJ. Whole exome sequencing with genomic triangulation implicates CDH2-encoded N-cadherin as a novel pathogenic substrate for arrhythmogenic cardiomyopathy. Congenit Heart Dis. (2017) 12:226–35. 10.1111/chd.12462 28326674

[44] van Hengel J Calore M Bauce B Dazzo E Mazzotti E Bortoli MD. Mutations in the area composita protein αT-catenin are associated with arrhythmogenic right ventricular cardiomyopathy. Eur Heart J. (2013) 34:201–10. 10.1093/eurheartj/ehs373 23136403

[45] Bermúdez-Jiménez FJ Carriel V Brodehl A Alaminos M Campos A Schirmer I. Novel desmin mutation p.Glu401Asp impairs filament formation, disrupts cell membrane integrity, and causes severe arrhythmogenic left ventricular cardiomyopathy&sol;dysplasia. Circulation. (2018) 137:1595–610. 10.1161/CIRCULATIONAHA.117.028719 29212896

[46] Ortiz-Genga MF Cuenca S Ferro MD Zorio E Salgado-Aranda R Climent V. Truncating FLNC mutations are associated with high-risk dilated and arrhythmogenic cardiomyopathies. J Am Coll Cardiol. (2016) 68:2440–51. 10.1016/j.jacc.2016.09.927 27908349

[47] Segura-Rodríguez D Bermúdez-Jiménez FJ Carriel V López-Fernández S González-Molina M Ramírez JMO. Myocardial fibrosis in arrhythmogenic cardiomyopathy: a genotype–phenotype correlation study. Eur Hear J Cardiovasc Imaging. (2019) 21:378–86. 10.1093/ehjci/jez277 31702781

[48] Taylor M Graw S Sinagra G Barnes C Slavov D Brun F. Genetic variation in titin in arrhythmogenic right ventricular cardiomyopathy–overlap syndromes. Circulation. (2011) 124:876–85. 10.1161/CIRCULATIONAHA.110.005405 21810661 PMC3167235

[49] Dominguez F Zorio E Jimenez-Jaimez J Salguero-Bodes R Zwart R Gonzalez-Lopez E. Clinical characteristics and determinants of the phenotype in TMEM43 arrhythmogenic right ventricular cardiomyopathy type 5. Heart Rhythm. (2020) 17:945–54. 10.1016/j.hrthm.2020.01.035 32062046

[50] Heijden JF van der Hassink RJ. The phospholamban p.Arg14del founder mutation in Dutch patients with arrhythmogenic cardiomyopathy. Neth Heart J. (2013) 21:284–5. 10.1007/s12471-013-0413-z 23595706 PMC3661881

[51] te Riele ASJM Agullo-Pascual E James CA Leo-Macias A Cerrone M Zhang M. Multilevel analyses of SCN5A mutations in arrhythmogenic right ventricular dysplasia/cardiomyopathy suggest non-canonical mechanisms for disease pathogenesis. Cardiovasc Res. (2017) 113:102–11. 10.1093/cvr/cvw234 28069705 PMC5220677

[52] Beffagna G Occhi G Nava A Vitiello L Ditadi A Basso C. Regulatory mutations in transforming growth factor-β3 gene cause arrhythmogenic right ventricular cardiomyopathy type 1. Cardiovasc Res. (2005) 65:366–73. 10.1016/j.cardiores.2004.10.005 15639475

[53] Zwaag PA Rijsingen IAW Asimaki A Jongbloed JDH Veldhuisen DJ Wiesfeld ACP. Phospholamban R14del mutation in patients diagnosed with dilated cardiomyopathy or arrhythmogenic right ventricular cardiomyopathy: evidence supporting the concept of arrhythmogenic cardiomyopathy. Eur J Heart Fail. (2012) 14:1199–207. 10.1093/eurjhf/hfs119 22820313 PMC3475434

[54] Merner ND Hodgkinson KA Haywood AFM Connors S French VM Drenckhahn J-D. Arrhythmogenic right ventricular cardiomyopathy type 5 is a fully penetrant, lethal arrhythmic disorder caused by a missense mutation in the TMEM43 gene. Am J Hum Genetics. (2008) 82:809–21. 10.1016/j.ajhg.2008.01.010 18313022 PMC2427209

[55] Buja G Nava A Daliento L Scognamiglio R Miorelli M Canciani B. Right ventricular cardiomyopathy in identical and nonidentical young twins. Am Heart J. (1993) 126:1187–93. 10.1016/0002-8703(93)90673-W 8237764

[56] Wlodarska EK Konka M Zaleska T Ploski R Cedro K Pucilowska B. Arrhythmogenic right ventricular cardiomyopathy in two pairs of monozygotic twins. Int J Cardiol. (2005) 105:126–33. 10.1016/j.ijcard.2004.11.016 16243102

[57] Akdis D Saguner AM Shah K Wei C Medeiros-Domingo A von Eckardstein A. Sex hormones affect outcome in arrhythmogenic right ventricular cardiomyopathy/dysplasia: from a stem cell derived cardiomyocyte-based model to clinical biomarkers of disease outcome. Eur Heart J. (2017) 38:1498–508. 10.1093/eurheartj/ehx011 28329361 PMC5837563

[58] Boese AC Kim SC Yin K-J Lee J-P Hamblin MH. Sex differences in vascular physiology and pathophysiology: estrogen and androgen signaling in health and disease. Am J Physiol Heart C. (2017) 313:H524–45. 10.1152/ajpheart.00217.2016 28626075 PMC5625178

[59] James CA Jongbloed JDH Hershberger RE Morales A Judge DP Syrris P. An international evidence based reappraisal of genes associated with arrhythmogenic right ventricular cardiomyopathy (ARVC) using the ClinGen framework. Circ Genom Precis Med. (2021) 14:273-84. 10.1161/CIRCGEN.120.003273 33831308 PMC8205996

[60] Ackerman MJ Priori SG Willems S Berul C Brugada R Calkins H. HRS/EHRA expert consensus statement on the state of genetic testing for the channelopathies and cardiomyopathies this document was developed as a partnership between the Heart Rhythm Society (HRS) and the European Heart Rhythm Association (EHRA). Heart Rhythm. (2011) 8:1308–39. 10.1016/j.hrthm.2011.05.020 21787999

[61] Miles C Finocchiaro G Papadakis M Gray B Westaby J Ensam B. Sudden death and left ventricular involvement in arrhythmogenic cardiomyopathy. Circulation. 139:1786–97. 10.1161/CIRCULATIONAHA.118.037230 30700137 PMC6467560

[62] Basso C Ronco F Marcus F Abudureheman A Rizzo S Frigo AC. Quantitative assessment of endomyocardial biopsy in arrhythmogenic right ventricular cardiomyopathy/dysplasia: an *in vitro* validation of diagnostic criteria. Eur Heart J. (2008) 29:2760–71. 10.1093/eurheartj/ehn415 18819962

[63] Corrado D Basso C Thiene G McKenna WJ Davies MJ Fontaliran F. Spectrum of clinicopathologic manifestations of arrhythmogenic right ventricular cardiomyopathy/dysplasia: a multicenter study. J Am Coll Cardiol. (1997) 30:1512–20. 10.1016/S0735-1097(97)00332-X 9362410

[64] Basso C Thiene G Corrado D Angelini A Nava A Valente M. Arrhythmogenic right ventricular cardiomyopathy: dysplasia, dystrophy, or myocarditis? Circulation. (1996) 94:983–91. 10.1161/01.CIR.94.5.983 8790036

[65] Chelko SP Asimaki A Lowenthal J Bueno-Beti C Bedja D Scalco A. Therapeutic modulation of the immune response in arrhythmogenic cardiomyopathy. Circulation. (2019) 140:1491–505. 10.1161/CIRCULATIONAHA.119.040676 31533459 PMC6817418

[66] van der Voorn SM te Riele ASJM Basso C Calkins H Remme CA van Veen TAB. Arrhythmogenic cardiomyopathy: pathogenesis, pro-arrhythmic remodelling, and novel approaches for risk stratification and therapy. Cardiovasc Res. (2020) 116:1571–84. 10.1093/cvr/cvaa084 32246823 PMC7526754

[67] Basso C Corrado D Marcus FI Nava A Thiene G. Arrhythmogenic right ventricular cardiomyopathy. Lancet. (2009) 373:1289–300. 10.1016/S0140-6736(09)60256-7 19362677

[68] Sen-Chowdhry S Morgan RD Chambers JC McKenna WJ. Arrhythmogenic cardiomyopathy: etiology, diagnosis, and treatment. Annu Rev Med. (2010) 61:233–53. 10.1146/annurev.med.052208.130419 20059337

[69] Avella A D'Amati G Pappalardo A Re F Silenzi PF Laurenzi F. Diagnostic value of endomyocardial biopsy guided by electroanatomic voltage mapping in arrhythmogenic right ventricular cardiomyopathy/dysplasia. J Cardiovasc Electr. (2008) 19:1127–34. 10.1111/j.1540-8167.2008.01228.x 18554207

[70] Pieroni M Russo AD Marzo F Pelargonio G Casella M Bellocci F. High prevalence of myocarditis mimicking arrhythmogenic right ventricular cardiomyopathy differential diagnosis by electroanatomic mapping-guided endomyocardial biopsy. J Am Coll Cardiol. (2009) 53:681–9. 10.1016/j.jacc.2008.11.017 19232901

[71] Casella M Bergonti M Russo AD Maragna R Gasperetti A Compagnucci P. Endomyocardial biopsy: the forgotten piece in the arrhythmogenic cardiomyopathy puzzle. J Am Heart Assoc. (2021) 10:e021370. 10.1161/JAHA.121.021370 34569251 PMC8649151

[72] Corrado D Link MS Calkins H. Arrhythmogenic right ventricular cardiomyopathy. N Engl J Med. (2017) 376:61–72. 10.1056/NEJMra1509267 28052233

[73] Towbin JA McKenna WJ Abrams DJ Ackerman MJ Calkins H Darrieux FCC. 2019 HRS expert consensus statement on evaluation, risk stratification, and management of arrhythmogenic cardiomyopathy. Heart Rhythm. (2019) 16:e301–72. 10.1016/j.hrthm.2019.05.007 31078652

[74] Kaplan SR Gard JJ Protonotarios N Tsatsopoulou A Spiliopoulou C Anastasakis A. Remodeling of myocyte gap junctions in arrhythmogenic right ventricular cardiomyopathy due to a deletion in plakoglobin (Naxos disease). Heart Rhythm. (2004) 1:3–11. 10.1016/j.hrthm.2004.01.001 15851108

[75] Kaplan SR Gard JJ Carvajal-Huerta L Ruiz-Cabezas JC Thiene G Saffitz JE. Structural and molecular pathology of the heart in Carvajal syndrome. Cardiovasc Pathol. (2004) 13:26–32. 10.1016/S1054-8807(03)00107-8 14761782

[76] Asimaki A Tandri H Huang H Halushka MK Gautam S Basso C. A new diagnostic test for arrhythmogenic right ventricular cardiomyopathy. N Engl J Med. (2009) 360:1075–84. 10.1056/NEJMoa0808138 19279339

[77] Noorman M Hakim S Kessler E Groeneweg JA Cox MGPJ Asimaki A. Remodeling of the cardiac sodium channel, connexin43, and plakoglobin at the intercalated disk in patients with arrhythmogenic cardiomyopathy. Heart Rhythm. (2013) 10:412–9. 10.1016/j.hrthm.2012.11.018 23178689 PMC3608196

[78] Nattel S Maguy A Bouter SL Yeh Y-H. Arrhythmogenic ion-channel remodeling in the heart: heart failure, myocardial infarction, and atrial fibrillation. Physiol Rev. (2007) 87:425–56. 10.1152/physrev.00014.2006 17429037

[79] Peters NS Green CR Poole-Wilson PA Severs NJ. Reduced content of connexin43 gap junctions in ventricular myocardium from hypertrophied and ischemic human hearts. Circulation. (1993) 88:864–75. 10.1161/01.CIR.88.3.864 8394786

[80] Kam CY Dubash AD Magistrati E Polo S Satchell KJF Sheikh F. Desmoplakin maintains gap junctions by inhibiting Ras/MAPK and lysosomal degradation of connexin-43. J Cell Biol. (2018) 217:3219–35. 10.1083/jcb.201710161 29959233 PMC6123000

[81] Asimaki A Tandri H Duffy ER Winterfield JR Mackey-Bojack S Picken MM. Altered desmosomal proteins in granulomatous myocarditis and potential pathogenic links to arrhythmogenic right ventricular cardiomyopathy. Circ Arrhyth Electrophysiol. (2011) 4:743–52. 10.1161/CIRCEP.111.964890 21859801 PMC3203520

[82] Rosset S Domingo AM Asimaki A Graf D Metzger J Schwitter J. Reduced Desmoplakin immunofluorescence signal in arrhythmogenic cardiomyopathy with epicardial right ventricular outflow tract tachycardia. Hear Case Rep. (2018) 5:57–62. 10.1016/j.hrcr.2018.06.013 30820396 PMC6379492

[83] Sato PY Musa H Coombs W Guerrero-Serna G Patiño GA Taffet SM. Loss of plakophilin-2 expression leads to decreased sodium current and slower conduction velocity in cultured cardiac myocytes. Circ Res. (2009) 105:523–6. 10.1161/CIRCRESAHA.109.201418 19661460 PMC2742576

[84] Cerrone M Noorman M Lin X Chkourko H Liang F-X van der Nagel R. Sodium current deficit and arrhythmogenesis in a murine model of plakophilin-2 haploinsufficiency. Cardiovasc Res. (2012) 95:460–8. 10.1093/cvr/cvs218 22764151 PMC3422082

[85] Asimaki A Kapoor S Plovie E Arndt AK Adams E Liu Z. Identification of a new modulator of the intercalated disc in a zebrafish model of arrhythmogenic cardiomyopathy. Sci Transl Med. (2014) 6:240ra74. 10.1126/scitranslmed.3008008 24920660 PMC4471875

[86] Cerrone M Lin X Zhang M Agullo-Pascual E Pfenniger A Gusky HC. Missense mutations in plakophilin-2 cause sodium current deficit and associate with a brugada syndrome phenotype. Circulation. (2014) 129:1092–103. 10.1161/CIRCULATIONAHA.113.003077 24352520 PMC3954430

[87] Kim C Wong J Wen J Wang S Wang C Spiering S. Studying arrhythmogenic right ventricular dysplasia with patient-specific iPSCs. Nature. (2013) 494:105–10. 10.1038/nature11799 23354045 PMC3753229

[88] Milstein ML Musa H Balbuena DP Anumonwo JMB Auerbach DS Furspan PB. Dynamic reciprocity of sodium and potassium channel expression in a macromolecular complex controls cardiac excitability and arrhythmia. Proc Natl Acad Sci USA. (2012) 109:E2134–43. 10.1073/pnas.1109370109 22509027 PMC3412015

[89] Petitprez S Zmoos A-F Ogrodnik J Balse E Raad N El-Haou S. SAP97 and dystrophin macromolecular complexes determine two pools of cardiac sodium channels Nav1.5 in cardiomyocytes. Circ Res. (2011) 108:294–304. 10.1161/CIRCRESAHA.110.228312 21164104

[90] Chelko SP Asimaki A Andersen P Bedja D Amat-Alarcon N DeMazumder D. Central role for GSK3β in the pathogenesis of arrhythmogenic cardiomyopathy. Jci Insight. (2016) 1:e85923. 10.1172/jci.insight.85923 27170944 PMC4861310

[91] Padrón-Barthe L Villalba-Orero M Gómez-Salinero JM Domínguez F Román M Larrasa-Alonso J. Severe cardiac dysfunction and death caused by ARVC type 5 is improved by inhibition of GSK3β. Circulation. (2019) 140:1188–204. 10.1161/CIRCULATIONAHA.119.040366 31567019 PMC6784777

[92] te Rijdt WP Asimaki A Jongbloed JDH Hoorntje ET Lazzarini E van Zwaag PA. Distinct molecular signature of phospholamban p.Arg14del arrhythmogenic cardiomyopathy. Cardiovasc Pathol. (2019) 40:2–6. 10.1016/j.carpath.2018.12.006 30763825

[93] Presland RB Dale BA. Epithelial structural proteins of the skin and oral cavity: function in health and disease. Crit Rev Oral Biol M. (2000) 11:383–408. 10.1177/10454411000110040101 11132762

[94] Asimaki A Protonotarios A James CA Chelko SP Tichnell C Murray B. Characterizing the molecular pathology of arrhythmogenic cardiomyopathy in patient buccal mucosa cells. Circ Arrhyth Electrophysiol. (2016) 9:e003688. 10.1161/CIRCEP.115.003688 26850880 PMC4785796

[95] Begay RL Graw SL Sinagra G Asimaki A Rowland TJ Slavov DB. Filamin C truncation mutations are associated with arrhythmogenic dilated cardiomyopathy and changes in the cell–cell adhesion structures. JACC Clin Electrophysiol. (2018) 4:504–14. 10.1016/j.jacep.2017.12.003 30067491 PMC6074050

[96] Voorn SVD Driessen H Lint FV Bourfiss M Mirzad F Onsri LE. Buccal mucosa cells as a diagnostic tool in patients with arrhythmogenic cardiomyopathy. Ep Europace. (2021) 23:euab116.114. 10.1093/europace/euab116.035 PMC8744793 35008484

[97] Chatterjee D Fatah M Akdis D Spears DA Koopmann TT Mittal K. An autoantibody identifies arrhythmogenic right ventricular cardiomyopathy and participates in its pathogenesis. Eur Heart J. (2018) 39:3932–44. 10.1093/eurheartj/ehy567 30239670 PMC6247665

[98] Caforio ALP Re F Avella A Marcolongo R Baratta P Seguso M. Evidence from family studies for autoimmunity in arrhythmogenic right ventricular cardiomyopathy. Circulation. (2020) 141:1238–48. 10.1161/CIRCULATIONAHA.119.043931 32114801

[99] Chatterjee D Pieroni M Fatah M Charpentier F Cunningham KS Spears DA. An autoantibody profile detects Brugada syndrome and identifies abnormally expressed myocardial proteins. Eur Heart J. (2020) 41:2878–90. 10.1093/eurheartj/ehaa383 32533187

[100] Calkins H. A new diagnostic test for arrhythmogenic right ventricular cardiomyopathy: is this too good to be true? Eur Heart J. (2018) 39:3945–6. 10.1093/eurheartj/ehy410 30239672

